# Anti-Inflammatory Activity of Methyl Salicylate Glycosides Isolated from *Gaultheria yunnanensis* (Franch.) Rehder

**DOI:** 10.3390/molecules16053875

**Published:** 2011-05-09

**Authors:** Dan Zhang, Rui Liu, Lan Sun, Chao Huang, Chao Wang, Dong-Ming Zhang, Tian-Tai Zhang, Guan-Hua Du

**Affiliations:** 1 National Center for Pharmaceutical Screening, Institute of Materia Medica, Chinese Academy of Medical Sciences & Peking Union Medical College, Beijing 100730, China; 2 Department of Phytochemistry, Institute of Materia Medica, Chinese Academy of Medical Sciences & Peking Union Medical College, Beijing 100730, China; 3 Key Laboratory of Bioactive Substances and Resources Utilization of Chinese Herbal Medicine, Ministry of Education, Beijing 100050, China

**Keywords:** Gaultheria plant, anti-inflammation, methyl salicylicate glycoside, inflammatory cytokines

## Abstract

*Gaultheria yunnanensis* (Franch.) Rehder is a kind of traditional Chinese herbal medicine used for the treatments of rheumatoid arthritis, swelling and pain. Two methyl salicylate glycosides, namely methyl benzoate-2-*O*-*β*-D-xylopyranosyl(1-6)-*O*-*β*-D-gluco-pyranoside (J12122) and methyl benzoate-2-*O*-*β*-D-xylopyranosyl(1-2)[*O*-*β*-D-xylopyranosyl(1-6)]-*O*-*β*-D-glucopyranoside (J12123), are natural salicylic derivatives isolated from *Gaultheria yunnanensis.* In this study, we investigated the anti-inflammatory activity of J12122 and J12123 on LPS-induced RAW264.7 macrophage cells by measuring the production of pro-inflammatory cytokines, accumulation of nitric oxide (NO), and level of reactive oxygen species (ROS). The results showed that both methyl salicylate glycosides dose-dependently inhibited the production of tumor necrosis factor-α (TNF-α), interleukin-1β (IL-1β), and IL-6, respectively. Consistent with these observations, J12122 and J12123 significantly suppressed the accumulation of NO, with an inhibitory rate of 56.20% and 51.72% at 3.0 μg/mL concentration, respectively. Furthermore, the two methyl salicylate glycosides reduced the level of ROS induced by LPS. These results showed that the isolated compounds possess anti-inflammatory properties through inhibition the production pro-inflammatory cytokines, NO, and ROS.

## 1. Introduction

Inflammation is a beneficial host response to foreign challenge or tissue injury that leads ultimately to the restoration of tissue structure and function [1]. In order to destroy the invading pathogens, numerous cellular and molecular factors are released against the invader, producing a attack on multiple levels [2,3,4]. However, like a “double edged sword”, inflammation, a pathogenesis of many disease states, may also be followed by prolonged inflammation [5]. During inflammatory processes, the recruitment of macrophages into the area of the infection, NO, reactive oxygen species (ROS), and cytokines such as TNF-α, IL-1β, and IL-6 are produced by the macrophages, and these are crucial mediators of inflammation response and tissue damage [6,7]. As a central mediator of inflammation, TNF-α is induced by a wide range of pathogenic stimuli and performs a key role in the pathogenesis of chronic inflammatory diseases [8,9]. Likewise, IL-1β is an important pro-inflammatory cytokine that regulates multiple aspects of immune and inflammatory responses. Acute inflammation induces the upregulation of IL-1β at sites of inflammation, and increased levels of IL-1β can provoke cell or tissue damage [10,11]. Additionally, IL-6 is a pivotal pro-inflammatory cytokine that mediates a plethora of physiological functions, including the developmental differentiation of lymphocytes, cell proliferation, and cell survival and amelioration of apoptotic signals [12,13,14,15]. Therefore, the level of pro-inflammatory factor was the important evaluating parameters for anti-inflammatory agents.

**Figure 1:**
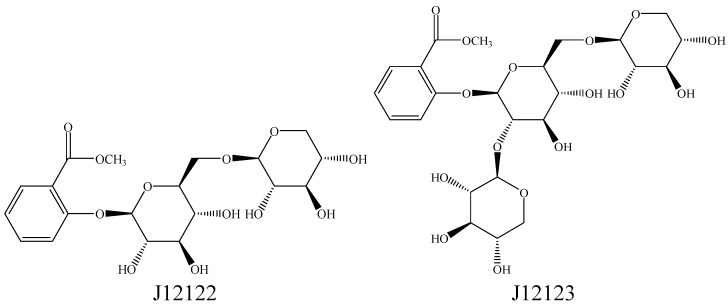
Structures of compounds J12122 and J12123 isolated from *G. yunnanensis.*

*Gaultheria yunnanensis* (Franch.) Rehder (*G. yunnanensis*), belongs to the Ericaceae family, and is a kind of traditional Chinese herbal medicine which grows in the southwest and southern regions of China and is widely used in these areas as a folk medicine for the treatments of rheumatoid arthritis, swellings, pain, trauma, chronic tracheitis, cold and vertigo [16]. Some salicylate derivatives, lignans, flavonoids, diterpenoids, triterpenoids, organic acids, coumarins, and sterols have been isolated from the roots of *G. yunnanensis* and identified [17]. We have extracted and isolated two methyl salicylate glycosides from the stems and leaves of *G. yunnanensis*. It is interesting to note that two compounds contain a chemical structure that is similar to that of salicylic acid. The two methyl salicylate glycosides, shown Figure 1, are methyl benzoate-2-*O*-*β*-D-xylopyranosyl(1-6)-*O*-*β*-D-glucopyranoside (J12122) and methyl benzoate-2-*O*-*β*-D-xylopyranosyl(1-2)[*O*-*β*-D-xylopyranosyl(1-6)]-*O*-*β*-D-gluco-pyranoside (J12123),. In this study, we aimed to investigate the anti-inflammatory activity of these two salicylate derivatives on lipopolysaccharide(LPS)-induced RAW264.7 cells. 

## 2. Results and Discussion

### 2.1. Compounds

The methyl salicylate glycosides were extracted from *G. yunnanensis* by the Department of Phytochemistry of our Institute. Briefly, the stems and leaves of *G. yunnanensis* were extracted three times with boiling 95% ethanol. After evaporation of ethanol *in vacuo*, the aqueous residue was diluted with water. The filtrate was separated macroreticular resin using water and 30% EtOH-water in sequence to afford fractions A_1_-A_2_. Part A_2_ was subjected to column chromatography on silica gel with CHCl_3_-MeOH-H_2_O (8:2:0.2) to afford methyl salicylate glycosides. After recrystallization with ethanol, two glycosides were obtained and identified as methyl benzoate-2-*O*-*β*-D-xylopyranosyl(1-6)-*O*-*β*-D-glucopyranoside (J12122) and methyl benzoate-2-*O*-*β*-D-xylopyranosyl(1-2)[*O*-*β*-D-xylo-pyranosyl(1-6)] -*O*-*β*-D-glucopyranoside (J12123).

We firstly examined the cytotoxic effects of J12122 and J12123 at several concentrations (0.1–100 μg/mL) using MTT assays. The results showed that neither compound affected the viability of RAW264.7 cells after 24 h incubation(data not shown).

### 2.2. Production of Pro-inflammatory Cytokines

**Figure 2:**
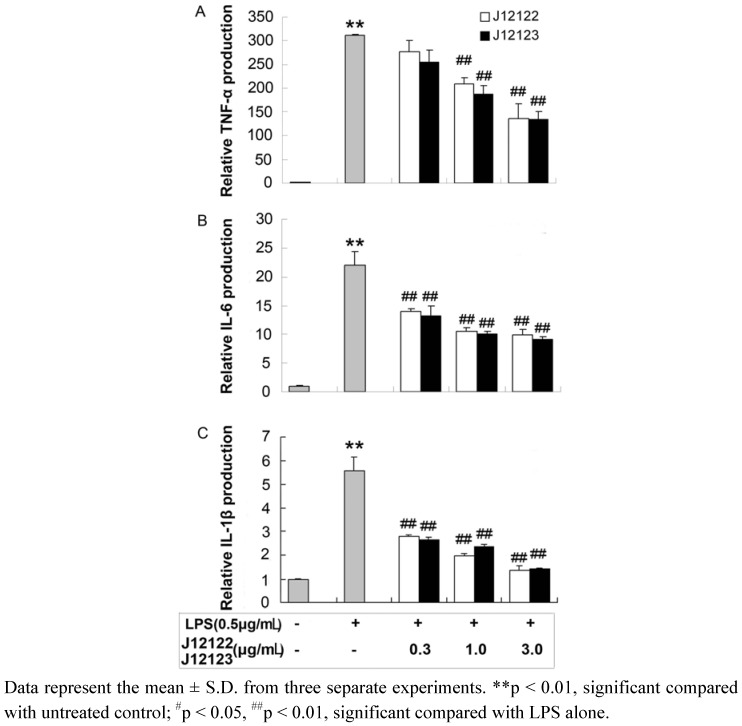
The effects of J12122 and J12123 on the production of TNF-α, IL-6 and IL-1β by LPS-induced RAW264.7 cells. (**A**) TNF-α, (**B**) IL-6 and (**C**) IL-1β contents in the culture medium, respectively.

Lipopolysaccharide (LPS), a prototypical endotoxin derived from Gram-negative bacterial membranes, can directly activate macrophages to trigger the production of pro-inflammatory mediators, such as TNF-α, IL-1β, and IL-6 [18,19]. LPS acts on macrophages to release TNF-α subsequently, the secreted TNF-α or LPS further induces the cells to release IL-1β and IL-6 [20]. Here, the production of the cytokines were measured by using ELISA assays on the media from the RAW264.7 cells treated with LPS (0.5 μg/mL) alone or those pre-treated with J12122 or J12123 in different concentrations. Figure 2 showed that the treatment of cells with LPS substantially increased the production of the cytokines within 12 h for TNF-α and IL-6, and within 18 h for IL-1β. Pre-incubated cells with J12122 or J12123 dose-dependently inhibited the production. The detailed inhibitory effect of J12122 and J12123 on pro-inflammatory cytokines was summarized in Table 1. The findings suggested that J12122 and J12123 all exerted an anti-inflammatory effect by suppressing the production of pro-inflammatory factors. This finding partly explain the results of our previous study that J12122 exerts a significant anti-inflammatory effect in croton oil-induced ear edema in mice [21].

**Table 1:** Inhibition of J12122 and J12123 on pro-inflammatory cytokines in RAW264.7 cells.

Pro-inflammatory cytokine	Inhibition of J12122 (%)			Inhibition of J12123 (%)		
	0.3 μg/mL	1.0 μg/mL	3.0 μg/mL	0.3 μg/mL	1.0 μg/mL	3.0 μg/mL
TNF-α	11.21 ± 2.08	32.69 ± 3.29	56.46 ± 2.98	18.37 ± 2.44	39.92 ± 3.09	57.16 ± 6.32
IL-1β	53.58 ± 3.56	64.40 ± 7.34	75.67 ± 8.02	54.47 ± 6.98	56.93 ± 4.51	74.33 ± 7.86
IL-6	61.81 ± 4.01	71.26 ± 6.46	73.15 ± 4.37	39.83 ± 5.42	53.92 ± 6.72	58.73 ± 6.78

Data represent the mean ± S.D. from three separate experiments.

### 2.3. Effects of NO Production

**Figure 3:**
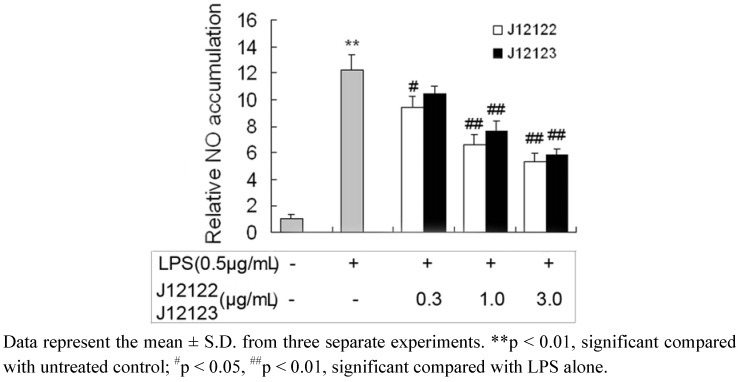
The inhibitory effects of J12122 and J12123 on LPS-stimulated NO accumulation.

As one of the important pro-inflammatory factors, NO plays a role in various physiological and pathophysiological conditions such as immunoregulatory and inflammatory processes [22]. Induction of NO synthesis has been identified as one of the major responses to inflammatory stimuli in macrophages by producing an excess amount of NO [23]. Although physiological NO production has beneficial microbicidal, anti-parasite and anti-tumour effects, excessive NO produced by iNOS is a mediator of inflammatory diseases and causes cell injury by generating reactive radicals [24,25,26]. To evaluate the effect of J12122 and J12123 on LPS-induced RAW264.7 cells, we examined the NO generation. The results indicated that LPS treatment increased NO production by 12-fold, as compared to control, which was significantly inhibited by J12122 or J12123 treatment (Figure 3). At the concentration of 1.0 μg/mL and 3.0 μg/mL, J12122 suppressed the accumulation of NO by 45.90% and 56.20% on RAW264.7 cells treated by LPS, likewise, J12123 was 37.45% and 51.72%, respectively.

### 2.4. Effect of Reactive Oxygen Species Production

**Figure 4:**
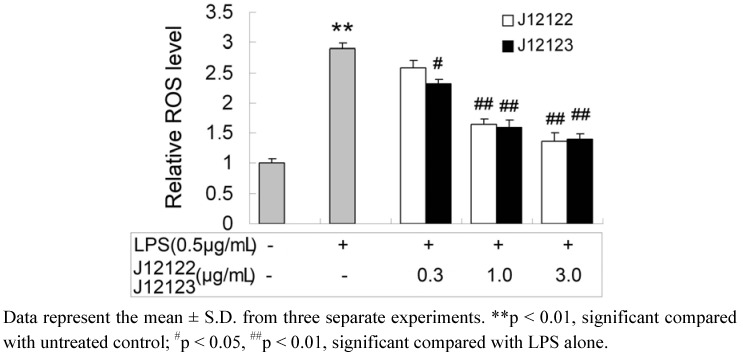
The inhibitory effects of J12122 and J12123 on LPS-stimulated ROS level.

ROS have been associated with the initiation or aggravation of diverse pathological states [27]. The excessive production of ROS by monocytes/macrophages at inflammatory sites affects the inflammation process [28]. Pro-inflammatory expression and NF-kB activation are strongly influenced by ROS [29]. Thus, ROS exerts a significant role in the development of inflammatory diseases. To determine the effect of J12122 and J12123 on intracellular ROS activated inflammation, the LPS-induced ROS level was detected in RAW264.7 cells in the presence or absence of J12122 or J12123. The production of ROS induced by LPS indicated a significant increase compared to LPS-untreated cells. As shown in Figure 4, LPS-induced ROS production in RAW264.7 cell was significantly inhibited by J12122 or J12123. 

## 3. Experimental

### 3.1. Chemical and Biological Materials

Unless otherwise specified, all chemicals were purchased from Sigma – Aldrich (St. Louis, MO, USA). Dulbecco's modified Eagle's medium (DMEM), fetal bovine serum and the antibiotic–antimycotic solution were purchased from Gibco (Aucland, NZ). The ELISA kits for TNF-α, IL-1β and IL-6 were obtained from Jiamei Biotech Co. (Beijing, China). Methyl benzoate-2-*O*-*β*-D-xylopyranosyl(1-6)-*O*-*β*-D-glucopyranoside (J12122) and methyl benzoate-2-*O*-*β*-D-xylopyranosyl(1-2)[*O*-*β*-D-xylopyranosyl(1-6)]-*O*-*β*-D-glucopyranoside (J12123) were extracted and isolated by the Institute of Materia Medica at the Chinese Academy of Medical Sciences.

### 3.2. Cell Culture

The RAW 264.7 murine macrophage cell line (ATCC TIB-71;American Type Culture Collection, Manassas, VA, USA) was cultured in Dulbecco's modified Eagle's medium (DMEM) supplemented with 10% heat-inactivated fetal bovine serum (FBS), 2 mM L-glutamine, 100 IU/mL penicillin and 100 μg/mL streptomycin in a 37 °C incubator with 5% CO_2_. For all experiments, the cells were grown to 80–90% confluence, with no more than 20 passages. Cells were stimulated by LPS (0.5 μg/mL) in the presence or absence of J12122 (0.3, 1.0, and 3.0 μg/mL) or J12123 (0.3, 1.0, and 3.0 μg/mL) for the measurement the production of pro-inflammatory mediator of TNF-α, IL-6, and IL-1β, NO, and ROS. Absorption value and fluorescent intensity were determined by using a microplate spectrofluorometer (Molecular Devices 5, Menlo Park, CA, USA). 

### 3.3. Plant Material

The plant material used in this study was collected from Dali, Yunnan Province, China, between April and September, in 2004. The plant was identified and authenticated by Dr. Chong-ren Yang, Kunming Institute of Botany, Chinese Academy of Sciences. A voucher specimen (No.Z040901) was made and deposited at the herbarium of the Institute of Materia Medica, Chinese Academy of Medical Sciences & Peking Union Medical College.

### 3.4. Cell Viability Assay

Cell viability was measured by a 3-(4,5-dimethylthiazol-2-yl)-2,5-diphenyltetrazolium bromide (MTT) assay, as previously described [30]. RAW 264.7 cells were seeded at a density of 10^4^ cells per well in a 96-well plate to determine any potential cytotoxicity. Cells were serum-starved for 12 h and then treated with J12122 or J12123 (0.1–100 μg/mL) for the next 24 h. The percentage of cell viability was calculated to evaluate the toxicity of J12122 and J12123 on RAW264.7 cells.

### 3.5. Enzyme-linked Immunosorbent Assay

Raw264.7 cells were cultured in 96-well plates (1 × 10^4^ cell/mL) and pre-incubated with J12122 (0.3, 1.0, and 3.0 μg/mL) or J12123 (0.3, 1.0, and 3.0 μg/mL) for 1 h and continuously incubated with LPS (0.5 μg/mL) for 12 h (TNF-α and IL-6) or for 18 h (IL-1β). TNF-α, IL-1β and IL-6 contents in the culture medium were measured by ELISA using anti-mouse TNF-α, IL-1β or IL-6 antibodies and a biotinylated secondary antibody, according to the manufacturer’s instructions. The optical density of each well was measured at 450 nm. The inhibition (%) was calculated using the following formula:

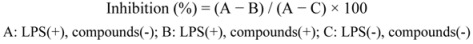


### 3.6. Measurement of NO Release (by Griess assay)

NO production was monitored by measuring the nitrite content in the culture medium. RAW 264.7 cells were pre-treated by J12122 or J12123 for 1 h and stimulated by LPS after 12 h of incubation. The NO concentration was quantified photometrically for the amount of stable nitrite produced in the cell culture supernatants using the Griess assay [31]. Each experiment was performed at least four times in duplicate and was compared with a standard curve plotted against different concentrations of sodium nitrite. The inhibition (%) was calculated using the following formula:

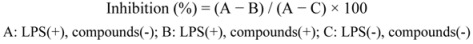


### 3.7. Production of Reactive Oxygen Species (ROS)

Intracellular ROS production was measured by incubating the cells with the fluorescent probe 2′,7′-dichlorodihydrofluorescein diacetate (DCFH-DA), as previously described [32]. RAW 264.7 cells were seeded (1 × 10^4^ cells/well) in 96- well plates and treated with increasing concentrations of J12122 or J12123 for 1 h before stimulation with LPS for 12 h. Then, DCFH-DA (10 μM) was added to incubate for an additional 45 min. Accumulation of dichlorofluorescein was measured as an increase in the fluorescence at 525 nm, when the samples were excited at 488 nm. The inhibition (%) was calculated using the following formula:

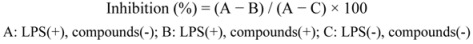


### 3.8. Statistical Analysis

Results are expressed as the mean ± S.D. of at least three experiments. Statistical significance was evaluated by the one-way ANOVA followed by the Student's t-test for paired populations. Differences among the means were assumed at a P value of 0.05 or less. 

## 4. Conclusions

In summary, our study demonstrated that the isolated methyl salicylate glycosides, natural salicylate derivatives from traditional Chinese herbs, exert anti-inflammatory activity, which results from the inhibition of pro-inflammatory cytokines (such as TNF-α, IL-1β and IL-6), NO and ROS production. Our findings showing inhibition of inflammation by these methyl salicylate glycosides may help to understand the primary pharmacological action and anti-inflammatory use of *Gaultheria yunnanensis* (Franch.) Rehder*.* More important, the findings presented here demonstrated that the methyl salicylate glycoside compounds might be potential anti-inflammatory leads. It also hints that the discovery of new anti-inflammatory leads, especially from natural products, may be a broad road.
